# Editorial comment – discovery,  post-genome  phylogenetic systematics, metabolites, function and application of cordycipitoid fungi

**DOI:** 10.1080/21501203.2017.1414743

**Published:** 2017-12-26

**Authors:** Zengzhi Li, Nigel Hywel-Jones, Chunru Li

**Affiliations:** Zhejiang BioAsia Life Science Institute, Zhejiang BioAsia Pharmaceutical, Ltd; Zhejiang BioAsia Life Science Institute; Zhejiang BioAsia Life Science Institutecrli@bioasia.com.cn

Cordycipitoid fungi, a large group of *Cordyceps sensu lato* species, contain more than 1000 species reported worldwide, some of them with a great value and application potential. New taxa are continuously being reported and the new classification system is constantly under revision. Genomics and metabolomics research is in the ascendant recently. At the same time, the constant search for new resources and valuable active ingredients in these treasures has always been a popular endeavour of researchers.

Pharmacological studies on cordycipitoid fungi have revealed that many constituents of these fungi and their fruiting bodies exert multifunctional pharmacological effects on endocrine, digestive, central nervous, cardiovascular and respiratory systems and reportedly to enhance the body’s resistance to inflammation and cancer. *Ophiocordyceps sinensis*, the most famous and valuable species, has been used as roborant and sedative for centuries. *Cordyceps militaris, Isaria cicadae* and *Cordyceps bassiana* also have attracted increasing attentions due to their multifunctions.

The recent worldwide Cordyceps forum once a year has promoted the academic exchanges and science popularisation in the field of cordycipitoid fungi research. In the era of international microbiology and healthy strategy background, the Cordyceps Forum 2016 conference placed a strong focus on the ongoing exploration of the genomics of *Cordyceps* and its innumerable hypocrealean relatives, both teleomorphic and anamorphic, as well as on the pharmacological capabilities of these fungi.

A Cordyceps special issue of *Mycology*, an international journal on fungal biology, is published in 2017. Some important reports presented in the 2016 Cordyceps Forum and research results of experts invited are reported in this special issue; key points are commented as follows.

Genomically based phylogenetic systematics is now effectively obligatory and enough genomic and molecular information has now accumulated about so many of these cordycipitoid fungi. However, Dr Humber rethinks that how much can we trust genomic data and how to interpret published genome sizes? … In this paper he indicated further that questions to be considered include how genomic information might help guide future research on such diverse issues as understanding the natural ecology of these fungi, the interactions of biotic and abiotic factors needed to initiate their sexual stages and better clues about how to elicit the productions of a wide range of biologically active compounds known to be produced by cordycipitoid fungi. This paper prospects direction of future research and thus adds value to the relevant problems.

Dr Sato reported the very important information about authentic type specimens of *Cordyceps* and allies described by Dr Kobayasi in the issue.

Dr Shrestha et al. introduced to the readers about current nomenclatural changes in *C. sensu lato* and its multidisciplinary impacts. Species compositions of the accepted genera *Ophiocordyceps, Tolypocladium, Metarhizium, Perennicordyceps, Polycephalomyces* and *Purpureocillium* are briefly discussed. Dr Nishi et al. showed us species associations and distributions of soil entomopathogenic fungi *Metarhizium* spp. in Japan. The papers update the readers with the current placements of *Cordyceps* spp and *Metarhizium* spp.

The fruiting body formation mechanisms of *Cordyceps sinensis* (≡*O. sinensis*) are still unclear. To explore the mechanisms, proteins potentially related to the fruiting body formation, proteins from fruiting bodies and mycelia of *Cordyceps* species were assessed by using two-dimensional fluorescence difference gel electrophoresis, and the differential expression proteins were identified by matrix-assisted laser desorption/ionisation tandem time of flight mass spectrometry (Feng et al.). The paper adds important experimental data to the rapidly growing literature.

Dr Cheng et al. confirmed myriocin in *I. cicadae* and its allies by LTQ-Orbitrap-HRMS. Meanwhile, the determination and comparative analysis of 13 nucleosides and nucleobases in natural fruiting body of *O. sinensis* and its substitutes were reported. Dr Dong et al. published a new N6-(2-hydroxyethyl)-adenosine–producing fungus – *Beauveria bassiana*. These results provided new information on relationship between the secondary metabolites and species of cordycipitoid fungi.

Prof. Li’s group analysed the radical scavenging active components in the fermented mycelia of *Ophiocordyceps formosana*. Dr Sun et al. reported that the conidia of *I. cicadae* (Chanhua) possess antiproliferative and induces apoptosis properties in gynaecological carcinoma cells. These findings add importance to the application of cordycipitoid fungi.

As summary, it is understood that cordycipitoid fungi have become an increasingly important source for various application. A wide variety of researches in the field of post-genome phylogenetic systematics, metabolites and function of cordycipitoid fungi have been popular lately, and therefore it is hoped that this special issue will help readers learn more and achieve more progress in the field of cordycipitoid fungi.

